# Stable and Unstable Malaria Hotspots in Longitudinal Cohort Studies in Kenya

**DOI:** 10.1371/journal.pmed.1000304

**Published:** 2010-07-06

**Authors:** Philip Bejon, Thomas N. Williams, Anne Liljander, Abdisalan M. Noor, Juliana Wambua, Edna Ogada, Ally Olotu, Faith H. A. Osier, Simon I. Hay, Anna Färnert, Kevin Marsh

**Affiliations:** 1 Kilifi KEMRI–Wellcome Trust Collaborative Research Programme, Centre for Geographic Medicine Research–Coast, Kilifi, Kenya; 2 Centre for Clinical Vaccinology and Tropical Medicine, University of Oxford, Churchill Hospital, Oxford, United Kingdom; 3 Department of Medicine Solna, Karolinska Institutet, Stockholm, Sweden; 4 Malaria Public Health & Epidemiology Group, Centre for Geographic Medicine Research–Coast, Kenya Medical Research Institute/Wellcome Trust Research Programme, Nairobi, Kenya; 5 Spatial Ecology and Epidemiology Group, Tinbergen Building, Department of Zoology, University of Oxford, Oxford, United Kingdom; Swiss Tropical Institute, Switzerland

## Abstract

**Background:**

Infectious diseases often demonstrate heterogeneity of transmission among host populations. This heterogeneity reduces the efficacy of control strategies, but also implies that focusing control strategies on “hotspots” of transmission could be highly effective.

**Methods and Findings:**

In order to identify hotspots of malaria transmission, we analysed longitudinal data on febrile malaria episodes, asymptomatic parasitaemia, and antibody titres over 12 y from 256 homesteads in three study areas in Kilifi District on the Kenyan coast. We examined heterogeneity by homestead, and identified groups of homesteads that formed hotspots using a spatial scan statistic. Two types of statistically significant hotspots were detected; stable hotspots of asymptomatic parasitaemia and unstable hotspots of febrile malaria. The stable hotspots were associated with higher average AMA-1 antibody titres than the unstable clusters (optical density [OD] = 1.24, 95% confidence interval [CI] 1.02–1.47 versus OD = 1.1, 95% CI 0.88–1.33) and lower mean ages of febrile malaria episodes (5.8 y, 95% CI 5.6–6.0 versus 5.91 y, 95% CI 5.7–6.1). A falling gradient of febrile malaria incidence was identified in the penumbrae of both hotspots. Hotspots were associated with AMA-1 titres, but not seroconversion rates. In order to target control measures, homesteads at risk of febrile malaria could be predicted by identifying the 20% of homesteads that experienced an episode of febrile malaria during one month in the dry season. That 20% subsequently experienced 65% of all febrile malaria episodes during the following year. A definition based on remote sensing data was 81% sensitive and 63% specific for the stable hotspots of asymptomatic malaria.

**Conclusions:**

Hotspots of asymptomatic parasitaemia are stable over time, but hotspots of febrile malaria are unstable. This finding may be because immunity offsets the high rate of febrile malaria that might otherwise result in stable hotspots, whereas unstable hotspots necessarily affect a population with less prior exposure to malaria.

*Please see later in the article for the Editors' Summary*

## Abstract 2

Philip Bejon and colleagues document the clustering of malaria episodes and malarial parasite infection. These patterns may enable future prediction of hotspots of malaria infection and targeting of treatment or preventive interventions.

## Editors' Summary

**Background:**

Malaria, a mosquito-borne parasitic disease, is a major global public-health problem. About half the world's population is at risk of malaria and about one million people (mainly children living in sub-Saharan Africa) die each year from the disease. Malaria is transmitted to people through the bite of an infected mosquito. Initially, the parasite replicates inside human liver cells but, about a week after infection, these cells release “merozoites” (one of the life-stages of the parasite), which invade red blood cells. Here, the merozoites replicate rapidly before bursting out after 2–3 days and infecting more red blood cells. The cyclical and massive increase in parasitemia (parasites in the bloodstream) that results from this pattern of replication is responsible for malaria's recurring fevers and can cause life-threatening organ damage and anemia (a lack of red blood cells). Malaria can be prevented by controlling the mosquitoes that spread the parasite and by avoiding mosquito bites. Effective treatment with antimalarial drugs can also reduce malaria transmission.

**Why Was This Study Done?:**

Like many other infectious diseases, the transmission of malaria is heterogeneous. That is, even in places where malaria is always present, there are “hotspots” of transmission, areas where the risk of catching malaria is particularly high. The existence of these hotspots, which are caused by a combination of genetic factors (for example, host susceptibility to infection) and environmental factors (for example, distance from mosquito breeding sites), reduces the efficacy of control strategies. However, mathematical models suggest that focusing control strategies on transmission hotspots might be an effective way to reduce overall malaria transmission. Efforts have been made to identify such hotspots using environmental data collected by satellites but with limited success. In this study, therefore, the researchers investigate the heterogeneity of malaria transmission in the Kilifi District of Kenya over time by analyzing data collected over up to 12 years (“longitudinal” data) on malaria episodes and parasitemia in three groups (cohorts) of children living in 256 homesteads.

**What Did the Researchers Do and Find?:**

The researchers identified febrile malaria episodes in the homesteads by taking blood from children with fever (febrile children) to analyze for parasitemia. They took blood once a year from all the study participants just before the rainy season (when malaria peaks) to look for symptom-free parasitemia and they also looked for antibodies (proteins made by the immune system that fight disease) against malaria parasites in the blood of the participants. They then used a “spatial scan statistic” to look for heterogeneity of transmission and to identify transmission hotspots (groups of homesteads where the observed incidence of malaria or parasitemia was higher than would be expected if cases were evenly distributed). The researchers identified two types of hotspots—stable hotspots of symptom-free parasitemia that were still hotspots several years later and unstable hotspots of febrile malaria that rarely stayed in the same place for more than a year or two. Children living in the stable hotspots had slightly higher average amounts of antimalaria antibodies and developed malaria at a slightly lower average age than children living in the unstable hotspots.

**What Do These Findings Mean?:**

These findings show that in Kilifi District, Kenya, hotspots of symptom-free parasitemia are stable over time but hotspots of febrile malaria are unstable. The researchers suggest that rapid acquisition of immunity in the stable hotspots reduces the occurrence of febrile malaria whereas in the unstable hotspots there is a high incidence of febrile malaria because lack of previous exposure to the parasite means there is a low level of immunity. Targeted strategies for malaria control should target both types of hotspots, suggest the researchers. Stable hotspots of symptom-free parasitemia (which can be identified by parasite or antibody surveys or by remote environmental sensing) should be targeted because mosquito dispersion probably increases malaria transmission rates near these hotspots. Unstable hotspots of febrile disease should be targeted to reduce both the burden of disease and transmission in the wider community. Unstable hotspots of febrile malaria, the researchers suggest, could be efficiently identified in Kilifi District (and maybe elsewhere) by determining which homesteads had malaria outbreaks during September (part of the dry season) one year and then focusing control interventions on these homesteads the next year.

**Additional Information:**

Please access these Web sites via the online version of this summary at http://dx.doi.org/10.1371/journal.pmed.1000304.

- Information is available from the World Health Organization on malaria (in several languages)
- The US Centers for Disease Control and Prevention provide information on malaria (in English and Spanish)
- MedlinePlus provides links to additional information on malaria (in English and Spanish)
- Information is available from the Roll Back Malaria Partnership on the global control of malaria (in English and French) and on malaria in Kenya

## Introduction

Many infectious disease show marked heterogeneity of transmission [1]. Mathematical models predict that this heterogeneity reduces the efficacy of disease control strategies [2], and intensifying control measures on foci of high transmission is predicted to be very effective in reducing overall transmission [1]. Marked spatial heterogeneity of malaria transmission on the household level is consistently detected when analysed [3]–[9], and results from both genetic and environmental factors [10],[11]. It is unclear how stable hotspots are in longitudinal data.

Malaria risk is related to environmental factors [12] including altitude [13], cultivation practices [14], urbanization [15], and distance from bodies of water [16]. However, ecological analyses to guide malaria control have been limited by the following factors: the overall models are complex [17]–[19], the same ecological feature may not have a consistent effect in different settings [20],[21], and there is marked residual variation in malaria risk despite models with detailed ecological data [22]. Furthermore, the resolution of multitemporal remote sensing satellite data (i.e. data based on more than a single snap-shot) for environmental monitoring is rarely finer than 0.5–1 km [23].

Since vector dispersion occurs on average over 0.5–1 km distances [24]–[29], this is the scale at which “hotspots” of transmission need to be identified in order to impact overall transmission. Malaria episodes have been found to cluster at this scale to form hotspots in high resolution geo-spatial analyses in Mali [6], Uganda [7], Ethiopia [30], and the highlands of Kenya [31],[32].

Here, we have conducted an analysis of malaria episodes and parasitaemia over 12 y, a substantially longer time period than has been reported previously, across three different cohorts without conspicuous variations in geography, such as nearby water bodies. We examine febrile episodes, asymptomatic parasitaemia, serological markers of exposure, environmental remote sensing data, and molecular studies of parasite clones to describe the spatial and temporal limits of hotspots, and to examine whether heterogeneity can be predicted.

## Methods

The approval for human participation in these cohorts was given by Kenya Medical Research Institute Scientific committee and National Review and Ethical Committee of Kenya Medical Research Institute, and was conducted according to the principles of the declaration of Helsinki.

### Surveillance for Malaria

**Figure 1:**
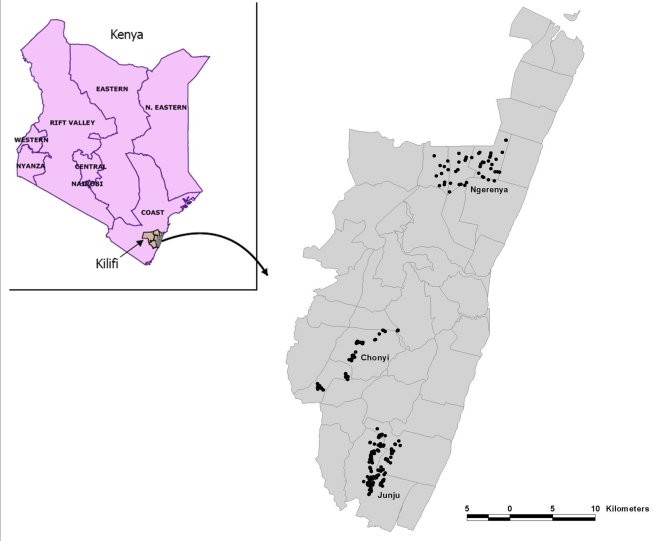
The distribution of homesteads monitored in the three cohorts is shown within Kilifi District.

The cohorts under surveillance for malaria episodes were located in Chonyi, Ngerenya, and Junju sublocations of Kilifi District, on the coast of Kenya between January 1998 and June 2009 (Figure 1). Concurrent entomological studies and parasite prevalence rates suggest that the transmission intensity is higher in Junju and Chonyi than in Ngerenya [33],[34], but transmission has been falling throughout the period of study [35].

The field methods used to identify episodes of febrile malaria and asymptomatic parasitaemia have previously been described [36],[37]. Weekly active surveillance was undertaken, and children with fever had blood slides for malaria parasites. In Chonyi and Ngerenya, children with either subjective (i.e., reported) or objective fever (temperature ≥37.5°C) had blood smears performed for estimating the parasite density. In Junju blood smears were done only on children with an objective fever, but children with subjective fever were seen again 6–12 h later, and the temperature measurement repeated. Blood smears were made if objective fever was confirmed at this measurement.

The parents of the children in Chonyi and Ngerenya were instructed to report to Kilifi District Hospital 20 km away if the child had any symptoms of disease at any time (and reimbursed for travel expenses), and in Junju trained field workers were available at all times in the villages for passive surveillance. Antimalarials were supplied for proven episodes of malaria by the study team in accordance with government of Kenya guidelines; this was sulfadoxine-pyrimethamine until 2004, and co-artemether thereafter. Study participants may have used private clinics or bought antimalarials without the study team's knowledge, but given the availability of free treatment this was probably infrequent.

Surveys for asymptomatic parasitaemia were undertaken once yearly, immediately before the rainy season. All individuals recruited to the study were asked to attend for blood sampling, and microscopy results were available for 70%–88% of participants for each survey.

The Geographic Positioning System coordinates from the Kilifi Demographic Surveillance Survey were linked to each homestead in the study. In Ngerenya and Chonyi, all the residents at individual homesteads were recruited, but in Junju only children under 8 y of age were recruited. The homesteads in Ngerenya and Junju were evenly spaced throughout the study location, but in Chonyi the homesteads were distributed along a central road through the study area. Children born in the study homesteads during the period of monitoring were recruited, and so the average age of the cohort did not increase over time (Table 1).

**Table 1:** Cohort summary characteristics.

Characteristics	Chonyi	Junju	Ngerenya
**Asymptomatic parasitaemia prevalence rate**	35%	32%	14%
**Incidence of febrile malaria (episodes per child year)**	0.82	0.55	0.49
**Average population**	818	462	428
**Median length of follow-up per child (y)**	2.8	3.5	5.0
**Median age of child (y)**	5.5	4.2	5.7
**Years of longitudinal monitoring in cohort**	3	5	11
**Homesteads**	59	149	48
**Participants per homestead**	13.9	3.1	8.7
**Average distance between adjacent sampled homesteads (km)**	0.08	0.08	0.35
**Age range (y)**	0–80	0–12	0–90
**Area including cohort dimensions N to S (km)**	7	7.6	6
**Area including cohort dimensions E to W (km)**	9.4	7.6	8.4

The asymptomatic parasitaemia prevalence includes both adults and children. The incidence of febrile malaria is given for participants <15 y age only.

### Definition of Febrile Malaria Incidence and Parasite Rates

Episodes of febrile malaria were determined using a parasite density threshold of >2,500/µl [36]. Episodes of febrile malaria were censored for 21 d after the last episode. The incidence per homestead per year was calculated by episodes of febrile malaria per homestead divided by the number of children who were <15 y old and by the number of years that participants were monitored. The parasite rate per homestead was calculated as the percentage of blood films positive for *Plasmodium falciparum* malaria from afebrile participants at scheduled blood sampling, including both adults and children.

### Analysis of Hotspots

The SaTScan software [38] was used to calculate the spatial scan statistic [39]. The spatial scan statistic uses a scanning window that moves across space. For each location and size of the window, the number of observed and expected cases is counted, and the window with the greatest ratio of observed to expected cases is noted. The numbers of expected cases are calculated by considering an even distribution of cases across the population. The statistical significance of this cluster (or “hotspot”) is then evaluated taking into account the multiple tests for the many potential cluster locations and sizes evaluated [40]. The scan statistic was then calculated for two types of cluster; to identify clusters (or “hotspots”) of febrile disease, using a discrete Poisson model; and to identify clusters (or “hotspots”) of asymptomatic parasitaemia, using a Bernoulli model, where cases were participants with parasitaemia and controls were participants without parasitaemia.

A spatial-only model was repeated each year rather than a temporal-spatial model for the entire period of monitoring for three reasons. First, the frequent seasonal peaks in transmission could only be adjusted for after assuming their uniformity across the study area. Secondly, the size of the database (11 y and 256 homesteads) made secondary clusters (or “hotspots”) very likely, and the option of excluding primary clusters to analyse for secondary clusters is not available for temporal-spatial analyses, but is validated for spatial-only analyses [41]. Thirdly, we could then compare hotspots of febrile disease on the basis of each year of monitoring with hotspots of asymptomatic parasitaemia and antibody titres measured on annual cross-sectional surveys.

The rate ratio (RR) was defined as the ratio of observed to expected cases, as produced in the SaTScan output. The test of significance was based on a Poisson generalized likelihood ratio test, using 9999 replications for a Monte Carlo inference. In order to run scans, the maximum cluster/hotspot size was set at 30% of the population, and the inference level for significance was set at 0.05 for primary clusters/hotspots (i.e., the window found with the highest RR). After removing the primary cluster/hotspot, the scan statistic was recalculated for the remaining locations in order to identify secondary clusters/hotspots. This calculation was only done where strongly significant (*p*<0.001) primary clusters/hotspots were identified.

A modified Poisson regression [42] was used to examine the relationships between clustering in different years, which were determined by scoring each homestead 0 or 1 for its absence or presence in any cluster under each condition.

### MSP-2 Genotyping of *P. falciparum* Infection

Amplification of the *merozoite surface protein 2 (MSP-2)* domain following capillary electrophoresis was used to accurately measure fragment size [43]. In the primary PCR reaction, the entire polymorphic region of *MSP-2* (block 3) was amplified, and in the following nested reactions the allelic types FC27 and IC were amplified separately. In the capillary electrophoresis-based method fluorescently labelled oligonucleotide primers were used in the nested reaction, and fragment analysis was performed in a 96-well format on a 3130xl DNA sequencer and GeneMapper Software version 4.0 (both Applied Biosystems). A 150 relative fluorescent unit (rfu) cut-off was used. Discrete clones were described by the allelic type and PCR product fragment size, aggregated over three base pair intervals (i.e., considered within the error of the method rather than the genetic structure of the alleles). Clones that were present in less than 3% of samples were described as “other.”

### Antibody Studies

Antibody titres against recombinant antigens of *P. falciparum* (3D7) were measured in a standard ELISA as reported in previous studies in Chonyi [44]–[46] and in Ngerenya [45],[46]. Antibody titres were not available from Junju. The recombinant antigen AMA-1 was provided by Sheetij Dutta and David Lanar [47], and recombinant MSP-2 was provided by Dave Conway and Spencer Polley using a plasmid provided by Jana McBride and David Cavanagh [46]. The optical density (OD) reading was used as a proxy for antibody concentration, and seropositivity was determined by an OD reading above the mean response plus three standard deviations for nonexposed control sera.

### Remote Sensing

MODIS is a sensor on board two NASA satellites. MODIS-derived temperature and reflectance layers were acquired for the years 2001 through 2008 [48]. A set of four channels; middle infra-red reflectance, daytime land surface temperature, night-time land surface temperature, and the enhanced vegetation index (EVI) were then processed by a temporal Fourier algorithm to achieve temporal ordination of the data time series while preserving important aspects of seasonal variation in the measures [49]. Each channel was then described by the following parameters: mean, minimum and maximum, and the amplitude, phase and variance of annual and bi-annual cycles decomposed from the data. A multivariable logistic regression model to predict hotspots using these parameters was developed from the best fit parameter in each channel, with subsequent exclusion of nonsignificant parameters (*p*>0.05). The most significant parameters from each channel were then combined in a final multivariable logistic regression model, with further exclusion of nonsignificant parameters (*p*>0.05). There are approximately 120 MODIS picture elements (pixels) widths/heights to each degree of latitude/longitude at the equator (i.e., approximately 1 km squared per pixel), so several homesteads are included in each pixel. The logistic regression models were therefore adjusted to take account of the nonindependence of observations by using the sandwich estimator to group observations by pixel [50]. The coefficients and constant from the final model (i.e., with *p*<0.05) were then used with the observed remote sensing data, to derive a predicted probability of a hotspot, which was then used to derive sensitivity and specificities for hotspot prediction.

## Results

Data were analysed from 256 homesteads in three study cohorts, where 5,600 episodes of febrile malaria were recorded over 32,452 person years of observation. The average incidence of malaria was 0.49 per child per year (cpy) in the lowest incidence cohort, and 0.82 per cpy in the highest incidence cohort (Table 1). Behind these summary statistics was substantial heterogeneity, with individual homestead incidences of febrile malaria ranging from zero to two episodes per cpy over the 3–11 y of monitoring (Figures S1 and S2).

### Spatial Limits of Clusters of Malaria

Episodes of febrile malaria were aggregated in “hotspots” or clusters (Table 2). The geometric mean of the RR for the clusters was 2.35 (range 1.3–28). 14% of the population monitored were within a hotspot at any one time. A total of 23 of the 26 hotspots were significant (i.e., *p*<0.05) and retained for further analysis. 17 of the 26 were strongly significant (i.e., *p*<0.005).

**Table 2:** Extent, degree, and significance of clustering of febrile malaria by location and year.

Year	Cluster or Hotspot	Radius (km)	Participant Population in Cluster/Total Cohort Population	Malaria Episodes in Cluster/All Malaria Episodes in Cohort	Population in Cluster (%)	Malaria Episodes in Cluster (%)	Rate Ratio of Malaria Episodes	*p*-Value
**Chonyi**								
**1999**	1st	2.8	201/776	233/673	26	35	1.34	0.0001
**2000**	1st	3.3	240/829	378/957	29	39	1.36	0.0001
**2001**	1st	2.3	163/848	153/466	19	33	1.71	0.0001
**Junju**								
**2005**	1st	0.9	13/349	19/214	4	9	2.38	0.013
**2005**	2nd	0.8	40/349	45/214	11	21	1.83	0.003
**2006**	1st	0.2	2/503	3/185	0	2	4.08	0.034
**2006**	2nd	1.1	129/503	68/185	26	37	1.43	0.01
**2007**	1st	1.4	131/452	84/206	29	41	1.41	0.033
**2008**	1st	2.1	97/400	177/481	24	37	1.52	0.001
**2009**	1st	0.6	19/376	12/53	5	23	4.48	0.003
**Ngerenya**								
**1998**	1st	2.5	90/394	45/143	23	31	1.38	0.0001
**1999**	1st	1.7	139/626	258/778	22	33	1.49	0.0001
**1999**	2nd	<0.1	9/626	26/778	1	3	2.32	0.0013
**1999**	3rd	<0.1	14/626	34/778	2	4	1.95	0.0006
**2000**	1st	1.7	145/643	333/851	23	39	1.74	0.0001
**2000**	2nd	<0.1	10/643	65/851	2	8	4.91	0.0001
**2001**	1st	2.4	154/700	264/755	22	35	1.59	0.0001
**2002**	1st	1.6	42/311	64/237	14	27	2.00	0.0001
**2002**	2nd	<0.1	7/311	17/237	2	7	3.19	0.0008
**2003**	1st	2.2	127/545	207/539	23	38	1.65	0.0001
**2003**	2nd	0.8	54/545	73/539	10	14	1.37	0.0003
**2003**	3rd	1.0	34/545	44/539	6	8	1.31	0.009
**2004**	1st	1.6	79/317	15/41	25	37	1.47	0.56
**2005**	1st	<0.1	2/319	3/17	1	18	28.15	0.0095
**2006**	1st	<0.1	7/345	2/9	2	22	10.95	0.43
**2007**	1st	0.7	28/320	3/6	9	50	5.71	0.32

*p*-Value given is for the significance of the cluster/hotspot, calculated from the Scan statistic and 9999 Monte Carlo randomizations. Hotspots are numbered in order of their identification using the scan statistic (the primary cluster always being first).

The average hotspot had a 1.3-km radius. There were six hotspots, where the apparent radius was less than the closest distance between sampled homesteads (i.e., the hotspot was a single homestead). All these hotspots were in Ngerenya, where there was the greatest distance between sampled homesteads (Table 1).

Asymptomatic parasitaemia was also aggregated into hotspots, with a geometric mean RR of 2.9 (range 1.5–15). 14% of the population monitored were within a hotspot. 19 of the 35 potential hotspots identified were significant (*p*<0.05) and retained for further analysis. Six were strongly significant (*p*<0.005). The average radius of these hotspots was 1 km.

### Gradient of Incidence on Moving from the Centre of a Hotspot to Outside the Hotspot

**Figure 2:**
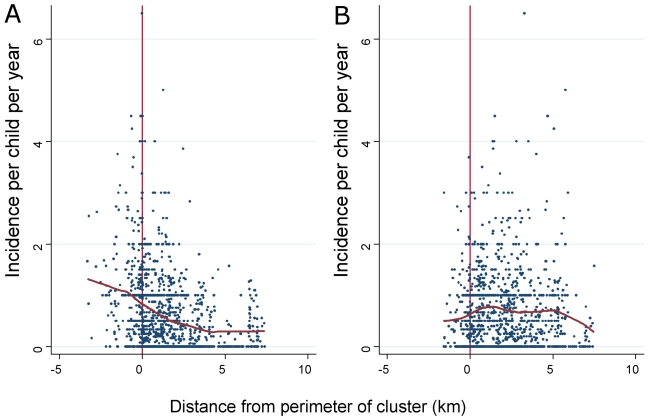
The incidence of febrile malaria for each homestead is plotted against distance from the perimeter of the cluster for (A) clusters of febrile malaria and (B) clusters of asymptomatic parasitaemia. Negative distances indicate a location inside the cluster, and positive distances indicate a location outside. Loess curves are shown in red (using a bandwidth of 0.8).

Based on the LOWESS curve, the incidence of febrile malaria was highest in the centre of a hotspot of febrile malaria (Figure 2), at 1.32 episodes/child year, and decreased towards the perimeter of the hotspot, where the mean incidence was 0.83 episodes/child year. The incidence of febrile malaria continued to fall gradually with distance outside the hotspot until reaching a plateau at 0.3 episodes/child year 3.7 km away from the perimeter of the hotspot. Between the centre to 3.7 km outside the perimeter, the gradient was −0.17 episodes/child year per km (95% confidence interval [CI] −0.2 to −0.14, *p* = 4×10^−21^). After 3.7 km, the gradient was −0.05 episodes/child year per km (95% CI −0.04 to 0.04, *p* = 0.8).

The highest point prevalence of asymptomatic parasitaemia was in the centre of a hotspot of asymptomatic parasitaemia, with a gradient of falling prevalence towards and then outside the perimeter of the hotspot. However, the incidence of febrile malaria was low in the centre of a hotspot of asymptomatic parasitaemia, at 0.49 episodes/child year by the LOWESS curve, and rose to 0.63 episodes/child year at the perimeter. Overall, the mean incidence within the hotspot was 0.6 episodes/child year, 95% CI 0.49–0.71. The peak incidence of 0.78 episodes/child year by LOWESS was reached 1.4 km away from the perimeter, and incidence then declined to 0.4 episodes/child year 7 km away from the perimeter. The mean incidence was 0.72 episodes/child year (95% CI 0.66–0.78) between 0 and 3 km from the perimeter of the hotspot of asymptomatic parasitaemia, and 0.48 episodes/child year (95% CI 0.41–0.55) after 3 km. The gradient of the incidence of febrile malaria was −0.033 episodes/child year per km outside the perimeter (95% CI −0.06 to −0.006, *p* = 0.016).

There was no consistent distance between contemporaneous hotspots of febrile malaria and hotspots of asymptomatic parasitaemia (range from 0 to 4.6 km).

### Malariometrics of Hotspots of Febrile Malaria Versus Hotspots of Asymptomatic Parasitaemia

Children within hotspots of asymptomatic parasitaemia had higher antibody titres on cross-sectional samples, and a lower mean age at the time of febrile malaria than children in hotspots of febrile malaria (Table 3). As a consequence of their respective definitions, the parasite rates were higher in hotspots of asymptomatic parasitaemia and the incidence of febrile malaria higher in hotspots of febrile malaria.

**Table 3:** Incidence, parasite rates, and antibody concentrations within clusters of asymptomatic parasitaemia and clusters of febrile malaria.

Characteristics	Overall	Hotspots of Asymptomatic Parasitaemia	Hotspots of Febrile Malaria
**Mean incidence of febrile malaria (95% CI)**	0.61 (0.57–0.65)	0.60 (0.48–0.71)	1.06 (0.94–1.18)
**Mean parasite rate (95% CI)**	25% (24–27)	48% (43–54%)	30% (27–33)
**Mean OD for antibody concentration (95% CI)**	0.96 (0.86–1.06)	1.24 (1.02–1.47)	1.1 (0.88–1.33)
**Mean age of febrile malaria (95% CI)**	5.17 (5.04–5.29)	4.89 (4.55–5.21)	5.05 (4.83–5.29)
**Mean age of all children monitored (95% CI)**	5.92 (5.81–6.03)	5.81 (5.62–5.98)	5.91 (5.73–6.08)

### Temporal Stability of Hotspots

**Figure 3:**
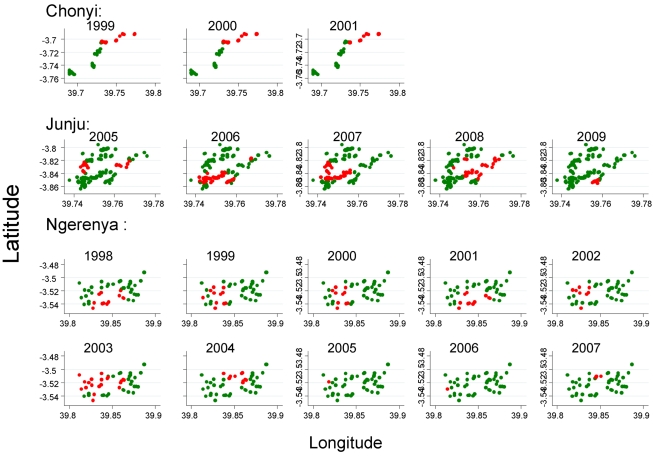
The distribution of homesteads in clusters of febrile malaria episodes (red), against homesteads outside the clusters (green) by year of monitoring for the three different cohorts.

**Figure 4:**
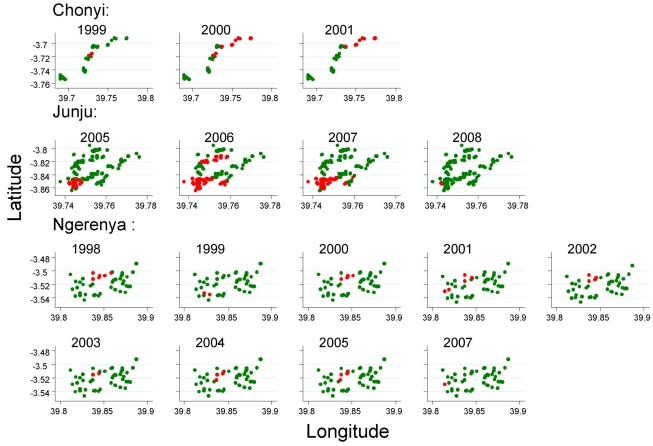
The distribution of homesteads in clusters of febrile malaria episodes (red), against homesteads outside the clusters (green) on a pooled analysis by season for the three different cohorts.

The position of hotspoting of febrile malaria changed from year to year in two study areas, although there was a subset of houses that remained within hotspots from year to year (Figure 3). Overall, the distribution of homesteads in a hotspot was moderately predictive of the distribution of hotspots for the following year, but became less predictive of the hotspots present after 4 y or longer. In contrast, hotspots of asymptomatic parasitaemia were more stable over time (Figure 4). The distribution of homesteads was more strongly predictive of hotspots the next year, and was still predictive of hotspots 7 y later (Table 4).

**Table 4:** Association between distribution of homesteads within a cluster over time intervals.

Interval between Clusters (y)	Febrile Malaria		Parasitaemia	
	Relative Risk	*p*-Value	Relative Risk	*p*-Value
**1**	2.71 (2.1–3.5)	2×10^−14^	4.47 (3.3–6.1)	2×10^−21^
**2**	2.04 (1.5–2.8)	8×10^−6^	3.61 (2.3–5.7)	2×10^−8^
**3**	2.90 (1.9–4.3)	2×10^−7^	8.56 (3.8–19)	2×10^−7^
**4**	1.19 (0.6–2.2)	0.59	11.6 (4.3–31)	1×10^−6^
**5**	0.72 (0.3–1.7)	0.47	10.9 (3.3–35)	7×10^−5^
**6**	0.22 (0.03–1.7)	0.15	4.64 (1.2–18)	0.03
**7**	0.84 (0.1–7)	0.88	14.5 (2.4–80)	0.003

**Figure 5:**
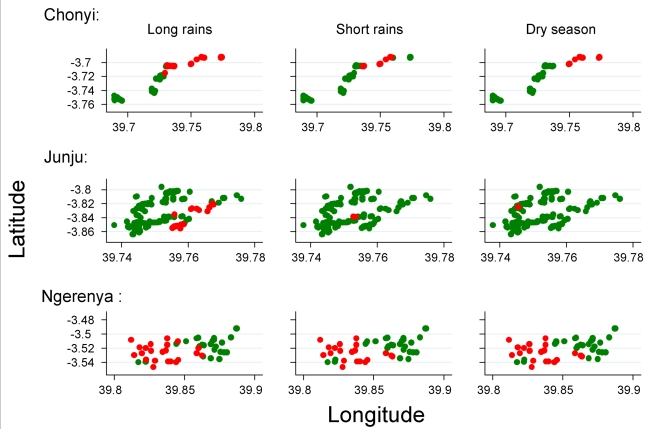
The distribution of homesteads in clusters of asymptomatic parasitaemia (red), against homesteads outside the clusters (green) by year of monitoring for the three different cohorts.

Although the location of hotspots of febrile malaria varied by year, they did not vary on a pooled analysis by season (Figure 5). The distribution of hotspots during the short rains predicted hotspots during the long rains (RR = 3.91, 95% CI 2.7–5.2, *p* = 7×10^−10^) and dry season (RR = 5.23, 95% CI 4.0–6.5, *p* = 3×10^−16^), and the hotspots seen in the dry season predicted hotspots during the long rains (RR = 4.45, 95% CI 3.0–5.9, *p* = 3.7×10^−9^).

### Homestead Level Clustering Versus Hotspots of Homesteads

There appeared to be substantial variation in the incidence of malaria outside the hotspots (Figures S2 and S3). Variation in risk at the homestead level was stable from year to year, even when analysing the homesteads inside and outside hotspots separately (Table 5). The geographical heterogeneity in asymptomatic parasitaemia at the homestead level was more stable over time than the heterogeneity for febrile malaria.

**Table 5:** Association between incidence and parasite rates by individual homesteads over time intervals.

Interval between Clusters (y)	Febrile malaria		Parasitaemia	
	Correlation Coefficient (*r*)	*p*-Value	Correlation Coefficient (*r*)	*p*-Value
**Overall**				
**1**	0.57	<0.00005	0.52	<0.00005
**2**	0.44	<0.00005	0.43	<0.00005
**3**	0.41	<0.00005	0.47	<0.00005
**4**	0.25	<0.00005	0.3	<0.00005
**5**	−0.12	0.03	0.29	<0.00005
**6**	−0.02	0.8	0.16	0.018
**7**	−0.06	0.33	0.1	0.22
**Inside cluster**				
**1**	0.4	<0.00005	0.37	<0.00005
**2**	0.24	0.026	0.38	0.016
**3**	0.37	0.003	0.64	0.001
**4**	0.2	0.15	0.2	0.45
**5**	−0.26	0.12	0.38	0.22
**6**	0.06	0.83	−0.14	0.7
**7**	−0.07	0.86	0.03	0.9
**Outside cluster**				
**1**	0.46	<0.00005	0.46	<0.00005
**2**	0.29	<0.00005	0.39	<0.00005
**3**	0.11	0.06	0.39	<0.00005
**4**	0.03	0.63	0.2	0.0016
**5**	−0.16	0.02	0.21	0.0039
**6**	−0.02	0.77	0.1	0.24
**7**	−0.07	0.41	−0.03	0.7

### Prediction of Hotspots by Serology

The antibody response to MSP-2 was used to determine hotspots in three ways; by the OD measurement (as a continuous variable), by the seropositivity rate, and by the seroconversion rate using a catalytic model. Neither the seropositivity rate nor seroconversion rate were associated with hotspots of febrile malaria or parasitaemia (OR = 0.99, 95% CI 0.98–1.01 *p* = 0.4 and OR = 1.03, 95% CI 0.95–1.1, *p* = 0.5). However, the OD measurements predicted concurrent hotspots of febrile malaria and parasitaemia significantly (OR = 3.77, 95% CI 1.3–10, *p* = 0.012 and OR = 5.9, 95% CI 1.7–20, *p* = 0.005, respectively), giving a sensitivity and specificity of 68% and 73% for febrile malaria hotspots, and 65% and 69% for asymptomatic parasitaemia hotspots, respectively. The OD remained associated with asymptomatic parasitaemia hotspots 3 y after the original measurement (OR = 20.1, 95% CI 1.2–300, *p* = 0.037), and with febrile malaria hotspots 2 y later (OR = 4.1, 95% CI 1.5–11, *p* = 0.005) but not at 3 y (OR = 2.5, 95% CI 0.3–18, *p* = 0.4).

AMA-1 responses were associated with hotspots of asymptomatic parasitaemia (OR = 3.5, 95% CI 1.3–10, *p* = 0.016) but not hotspots of febrile malaria (OR = 1.5, 95% CI 0.7–3.5, *p* = 0.3) (See Figure S1 for receiver operating characteristic [ROC] curves for antibody titres).

### Prediction of Hotspots by Remote Sensing Data

Environmental factors indicated by remote sensing data were strongly implicated in determining hotspots of asymptomatic parasitaemia, either by selection of dominant parameters or from principal component analysis (Table 6). The first three components of the principal component analysis had Eigenvalues of 7.09, 5.97, and 4.43, accounting for 49% of the total variance. The remaining components had Eigenvalues <3, and each accounted for 8% or less of the total variance. Definitions based on the logistic regression models from the MODIS satellite data were 65% sensitive and 67% specific from principal component analysis or 81% sensitive and 63% specific based on the model of selected independent predictors.

**Table 6:** Association between proportion of homesteads in a hotspot and environmental remote sensing data.

Variable	Odds Ratio (95% CI)	*p*-Value
***Hotspots of asymptomatic parasitaemia***		
**Chonyi**	1	—
**Junju**	1.08 (0.34–3.5)	0.9
**Ngerena**	0.97 (0.30–3.1)	0.9
**Enhanced vegetation index: min**	0.07 (0.03–0.2)	3×10^−7^
**Daytime temp (°C): bi-annual phase**	0.13 (0.05–0.3)	4×10^−6^
**Middle infra-red: bi-annual phase**	5.9 (1.6–22)	0.0085
**Night-time temp (°C): max**	0.03 (0.003–0.25)	0.0015
**Principal components**		
**First**	0.99 (0.82–1.2)	0.92
**Second**	0.36 (0.15–0.86)	0.021
**Third**	1.46 (1.02–2.1)	0.037
***Hotspots of febrile malaria episodes***		
**Chonyi**	1	—
**Junju**	0.84 (0.26–2.7)	0.8
**Ngerena**	0.53 (0.16–1.7)	0.3
**Middle infra-red, annual variance**	0.9 (0.85–0.95)	1.3×10^−4^
**Principal components**		
**First**	0.72 (0.55–0.95)	0.021
**Second**	0.86 (0.59–1.3)	0.8
**Third**	1.26 (0.86–1.9)	0.2

Results from four separate models: multivariable logistic regression was done to predict hotspots of asymptomatic b (a) individual variables and (b) principal component analysis, and to predict hotspots of febrile malaria episodes b (a) individual variables and (b) principal component analysis.

Hotspots of febrile malaria were less strongly linked to the remote sensing data, and the significant factors differed from those that identified hotspots of asymptomatic parasitaemia. Definitions for febrile malaria hotspots were only 62% sensitive and 30% specific from principal components or 81% sensitive and 50% specific from the selected independent predictor (see Figure S3).

### Parasite Genotyping Data by Hotspot

In Junju parasite genotypes according to *MSP-2* fragment size by capillary tube electrophoresis were available. There were 26 common alleles (i.e., represented in more than ten individuals) and 66 less common alleles that were classified as “other”. An overall analysis of variance showed significant variation in the distribution of clones (*p* = 0.016). Post hoc testing (Table 7) suggested that this variation was due to three genotypes that were overrepresented inside the hotspot: *fc419* (16% versus 8%, *p* = 0.004); *ic496* (5% versus 2%, *p* = 0.015); and *ic545* (4% versus 1%, *p* = 0.017).

**Table 7:** Distribution of parasite genotype b location inside versus outside a hotspot of asymptomatic parasitaemia.

Genotype	Frequency in Hotspot, *n* (%)	Frequency Outside Hotspot, *n* (%)	*p*-Value
***fc299***	9 (6)	35 (4)	0.3
***fc308***	1 (0)	20 (2)	0.3
***fc336***	17 (12)	111 (15)	0.98
***fc372***	7 (5)	60 (8)	0.6
***fc382***	4 (2)	18 (2)	0.7
***fc419***	22 (15)	55 (7)	0.004
***fc479***	3 (2)	7 (0)	0.4
***ic460***	4 (2)	10 (1)	0.14
***ic469***	0 (0)	13 (1)	—
***ic478***	5 (3)	25 (3)	0.5
***ic484***	1 (0)	14 (1)	0.5
***ic487***	6 (4)	24 (3)	0.4
***ic491***	4 (2)	14 (1)	0.2
***ic496***	7 (5)	13 (1)	0.015
***ic504***	4 (2)	26 (3)	0.9
***ic507***	3 (2)	10 (1)	0.4
***ic516***	2 (1)	22 (3)	0.4
***ic522***	4 (2)	20 (2)	0.5
***ic533***	0 (0)	15 (2)	—
***ic538***	3 (2)	16 (2)	0.9
***ic545***	6 (4)	10 (1)	0.017
***ic556***	2 (1)	12 (1)	0.9
***ic559***	1 (0)	19 (2)	0.3
***ic581***	0 (0)	17 (2)	—
***ic591***	3 (1)	7 (0)	0.4
***ic598***	2 (1)	9 (1)	0.9
***Other, low frequency***	21 (15)	128 (17)	—

An overall analysis of variance showed *p* = 0.016. Individual *p*-values are shown for post hoc testing of each genotype's distribution.

### Predicted Operational Performance of Predictors of Heterogeneity

Using antibody titres, 20% of homesteads would be identified for an intervention with a cut-off of mean titre >1.0 OD, but only 37% of the whole cohort's malaria episodes would occur in these homesteads. In order to include 50% of the malaria episodes, a cut-off of 0.85 would be required, and this would require 32% of the homesteads in the study area to be treated.

Table 8 shows the results of monitoring episodes of febrile malaria during 1 or 2 mo of surveillance to predict febrile malaria episodes during the following year, on the basis of the assumption that any homestead with at least one episode of febrile malaria during the monitoring period would be targeted for treatment.

**Table 8:** Predicted performance if all homesteads with one or more episode of febrile malaria during 1-mo monitoring are targeted for measures to interrupt transmission over the following year.

Monitoring	Percent Homesteads Targeted	Percent Malaria Covered
**January (short rains)**	28	73
**March (dry)**	17	48
**May (start long rains)**	26	68
**September (dry)**	20	65
**November (dry)**	17	56

## Discussion

We identified stable hotspots of asymptomatic parasitaemia, and unstable hotspots of febrile disease in each of three cohorts in Kilifi District. The hotspots of asymptomatic parasitaemia were characterized by nonsignificantly higher mean antibody titres, a lower mean age at febrile episodes, and lower overall incidences of febrile disease. There may have been an increase in the incidence of febrile malaria in the penumbra around the perimeter of the hotspots of asymptomatic parasitaemia (Figure 5). Furthermore, hotspots of asymptomatic parasitaemia were stable over the full 7 y of monitoring, whereas hotspots of febrile malaria were not stable past 3 y of monitoring. Taken together, these observations suggest the following explanation: that a rapid acquisition of immunity in stable high transmission hotspots offsets the high rates of febrile malaria that would otherwise result. Instead, a high prevalence of asymptomatic parasitaemia is seen [51]. Unstable hotspots are not associated with prior exposure, and hence relatively low levels of immunity, and therefore produce higher incidences of febrile disease. Although the unstable hotspots are directly associated with more febrile disease, hotspots of asymptomatic parasitaemia may be critical in maintaining transmission [52].

The satellite remote sensing data suggest a primarily environmental cause for stable hotspots, but it was unclear why some hotspots are unstable. It was not simply a seasonal effect, since there was no indication of a seasonal specificity of hotspots in pooled analysis by season. Potential explanations might be a changing prevailing wind direction [53] or the temporary presence of “superspreaders” as seen in other infectious diseases [54]. Insecticide-treated net (ITN) use has expanded in Kilifi generally [35] and in the Junju cohort specifically [55], but there was no evidence that there were hotspots of ITN use in our dataset (*p* = 0.9).

The study is based on active case detection in a sample of the total population (5%–10%). Data from the missing homesteads might alter the dimensions and frequency of the hotspots identified, and clarify whether clustering is stronger at the level of individual homestead or at the level of groups of homesteads that form “hotspots.” The resolution of remote sensing data results in an average of six homesteads per pixel. The remote sensing data predicted hotspots, but heterogeneity in risk by individual homestead was also observed, and higher resolution remote sensing data are needed to further investigate this.

The instability of hotspots of febrile malaria was not simply due to variations in age of the children monitored (Table 3). Furthermore, stable hotspots of asymptomatic parasitaemia were observed in Junju, where only children and not adults were recruited to the cohort. Although the three cohorts monitored were from geographically different areas, the study is limited by presenting data from a single district, and so cannot represent the great diversity of ecology that will be seen across sub-Saharan Africa. Other cohorts undergoing longitudinal surveillance should be examined to confirm our findings.

Malaria transmission varies by geographical features such as altitude [13], cultivation practices [14], streams and dams [16], house construction [56], socioeconomic factors [57], and ITN use [58]. Data on these factors were not available for the analysis presented here, although there were no large water bodies in the study area. However, a previous analysis in one of the study areas demonstrated that both environmental factors at the homestead level and host genetic factors contributed substantially to variation in malaria risk [10].

Our analysis suggests that environmental factors identified by remote sensing are associated with stable hotspots of asymptomatic parasitaemia. The environment in Kenya is seasonal, with two rainy seasons per year. The strongest individual factors from remote sensing were not the means of any index, but rather the minimum, and phases for a vegetation index and indicators of temperature, consistent with previous studies that have demonstrated the importance of temporal monitoring [12]. The remote sensing data show strong cross-correlation, and significant individual factors are likely to be proxies for more complex environmental determinants of transmission. 48 different measures of remote sensing were tested, but after a Bonferroni correction *p* = 1.4×10^−5^ and *p* = 2×10^−4^ for the two most significant individual factors.

The evidence for environmental causation of the unstable hotspots of febrile malaria was less strong. Nevertheless, *p* = 0.006 after a Bonferroni correction for the single individual factor retained in the final model.

On principle component analysis the second and third components were significantly associated with hotspots of asymptomatic parasitaemia, but the first component was associated with hotspots of febrile malaria. This finding is consistent with a complex environmental causation of hotspots rather than a single factor, and different environmental causes for the two types of hotspot.

Anti-AMA antibody titres were associated with hotspots, but seroprevalence and seroconversion rates did not predict hotspots. This association contrasts with previous findings on a larger geographical scale [59]. The effects of individual variation may become more noticeable on a smaller scale [60], and smaller scale hotspots are unlikely to be as stable as environmental features such as altitude that operate on a larger scale [61].

There was substantial heterogeneity of transmission inside and outside hotspots. This heterogeneity was not simply random variation, since the previous year's incidence per homestead was strongly predictive of subsequent risk (*r* = 0.57). Variation in malaria risk at the homestead level has been shown to be due to a mixture of genetic and environmental factors [10],[11]. Irrespective of the cause, hotspots have a substantial effect on overall transmission in the community [2]. Here, we demonstrate two levels of clustering; clustering at the homestead level, and hotspots of groups of high risk homesteads in 1.3-km radius areas. The “gradient” effect away from the perimeter of a hotspot is consistent with transmission in the majority of the cohort being maintained by transmission from within the hotspot of homesteads. Hence, targeting the 73% of febrile malaria within the 28% highest risk homesteads is likely to benefit the wider community, since the highest risk homesteads will increase transmission in the surrounding area [1].

The genotyping data suggest that individual parasite clones are associated with a hotspot of asymptomatic parasitaemia. This finding may be because particular hosts more frequently transmit their parasites within a hotspot [54], or because particular parasite clones have adapted to a geographical micro-environment, determined, for instance, by the vector species [62]. Genotypes were not available from febrile disease episodes.

Clustering of episodes by individual is reported in many infectious diseases [1], and has been relevant to the control of diverse pathogens such as *Escherichia coli* [54], tuberculosis [63], gonorrhea [64], SARS [65], and leishmaniasis [66]. Clustering of malaria episodes by homestead [10] and larger geographical hotspots including groups of homesteads is well described [3]–[9],[67], but there are few data on the temporal stability of hotspots.

Targeted strategies for malaria control need to consider two kinds of hotspot; stable hotspots of asymptomatic parasitaemia and unstable hotspots of febrile disease. One might argue against intervening in hotspots of asymptomatic parasitaemia, since rates of febrile disease are not high, and intervention might reduce host immunity. However, where transmission has fallen in areas of high transmission, substantial direct and indirect mortality and public health gains have been described in the short and long term [35],[68],[69]. Furthermore, these stable hotspots probably feed transmission in their penumbrae for a distance of several kilometres because of vector dispersion [24]–[29]. Hotspots of asymptomatic parasitaemia can be identified by parasite surveys, serological surveys, or, more conveniently, remote sensing.

Hotspots of febrile disease may be targeted by monitoring presentations to the local dispensary during the dry season, and targeting the effected homesteads during the subsequent rains. The optimal protocol would be to monitor during the dry season in September and then treat for the following year, which would result in targeting 20% of the homesteads, accounting for 65% of the febrile malaria episodes during the following year. It is optimal to cover 100% of the homesteads with any control intervention (particularly ITN distribution). However, some other interventions are currently not practical on community-wide scale, such as repeated mass drug administration [70],[71], environmental modification [72]–[74], mass vaccination [75], or (in some circumstances) indoor residual spraying (IRS) [76]–[78], but become feasible if targeted on the 20% of homesteads at greatest risk.

When transmission has reduced to very low levels, transmission remains geographically clustered [9],[79], and intensified control in these hotspots is key to achieving elimination. In our setting, as in much of sub-Saharan Africa, there is not an immediate prospect of elimination. Additional targeted control measures may be viewed simply as a cost-effective way of ensuring that those most in need get the intervention, but the stronger justification is that reducing transmission in hotspots will reduce transmission on the wider community [1].

Environmental interactions are complex in determining malaria risk per se [12], and so these findings should be validated in other datasets before firm recommendations for malaria control programmes are made.

## Supporting Information

Figure S1 Receiver operator characteristics (ROCs) are shown for AMA-1 antibodies, MSP-2 antibodies, and for the model on the basis of selected remote sensing variables and principle component analysis of the remote sensing variables. The areas under the ROC curves were 0.61, 0.67, 0.68, and 0.68 for prediction of febrile malaria hotspots by AMA-1 and MSP-2 antibodies, and for the model and principal component analyses, respectively. For asymptomatic parasitaemia hotspots the areas under the curves were 0.70, 0.72, 0.82, and 0.73, respectively.
(0.61 MB TIF)

Figure S2 The incidence of febrile malaria episodes cpy is shown for the three cohorts. More intense green colouring indicates higher incidence.
(0.48 MB TIF)

Figure S3 The prevalence of asymptomatic malaria is shown for the three cohorts. More intense green colouring indicates higher incidence.
(0.47 MB TIF)

## Abbreviations

CI: confidence interval
cpy: child per year
ITN: insecticide-treated net
OD: optical density
RR: rate ratio
