# Correction: Stable and Unstable Malaria Hotspots in Longitudinal Cohort Studies in Kenya

**DOI:** 10.1371/annotation/592709c8-8f86-4f40-968b-1e7e051ab491

**Published:** 2011-02-24

**Authors:** Philip Bejon, Thomas N. Williams, Anne Liljander, Abdisalan M. Noor, Juliana Wambua, Edna Ogada, Ally Olotu, Faith H. A. Osier, Simon I. Hay, Anna Färnert, Kevin Marsh

In the published article the part of the funding statement describing how Philip Bejon was supported should read: "PB is supported by the NIHR Biomedical Research Centre in Oxford." The corrected funding statement for this article reads in full: &ldquo;The study was supported by the Wellcome Trust and by the Kenya Medical Research Institute (KEMRI). PB is supported by the NIHR Biomedical Research Centre in Oxford. KM, TNW, and FO are supported by the Wellcome Trust. SIH is funded by a Senior Research Fellowship from the Wellcome Trust (#079091). The funders had no role in study design, data collection and analysis, decision to publish, or preparation of the manuscript.&rdquo; 

