# Supplementary figures and images for: Stable and Unstable Malaria Hotspots in Longitudinal Cohort Studies in Kenya
Source: PLoS Med. 2010 Jul 6;7(7):e1000304. doi: 10.1371/journal.pmed.1000304 (PMC2897769; doi:10.1371/journal.pmed.1000304)

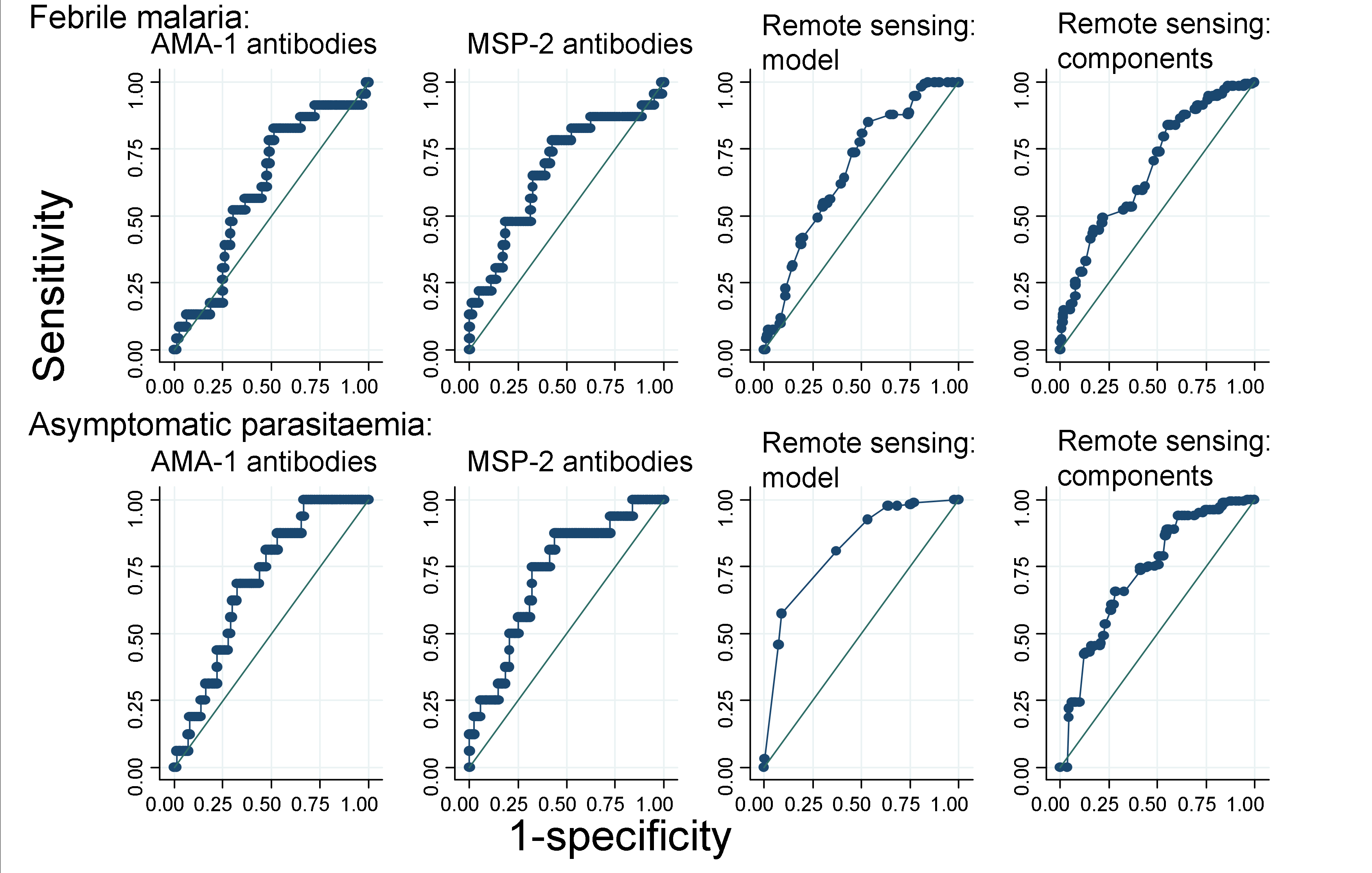

Supplement: Figure S1 — Receiver operator characteristics (ROCs) are shown for AMA-1 antibodies, MSP-2 antibodies, and for the model on the basis of selected remote sensing variables and principle component analysis of the remote sensing variables. The areas under the ROC curves were 0.61, 0.67, 0.68, and 0.68 for prediction of febrile malaria hotspots by AMA-1 and MSP-2 antibodies, and for the model and principal component analyses, respectively. For asymptomatic parasitaemia hotspots the areas under the curves were 0.70, 0.72, 0.82, and 0.73, respectively. (0.61 MB TIF) [file pmed.1000304.s001.tif]

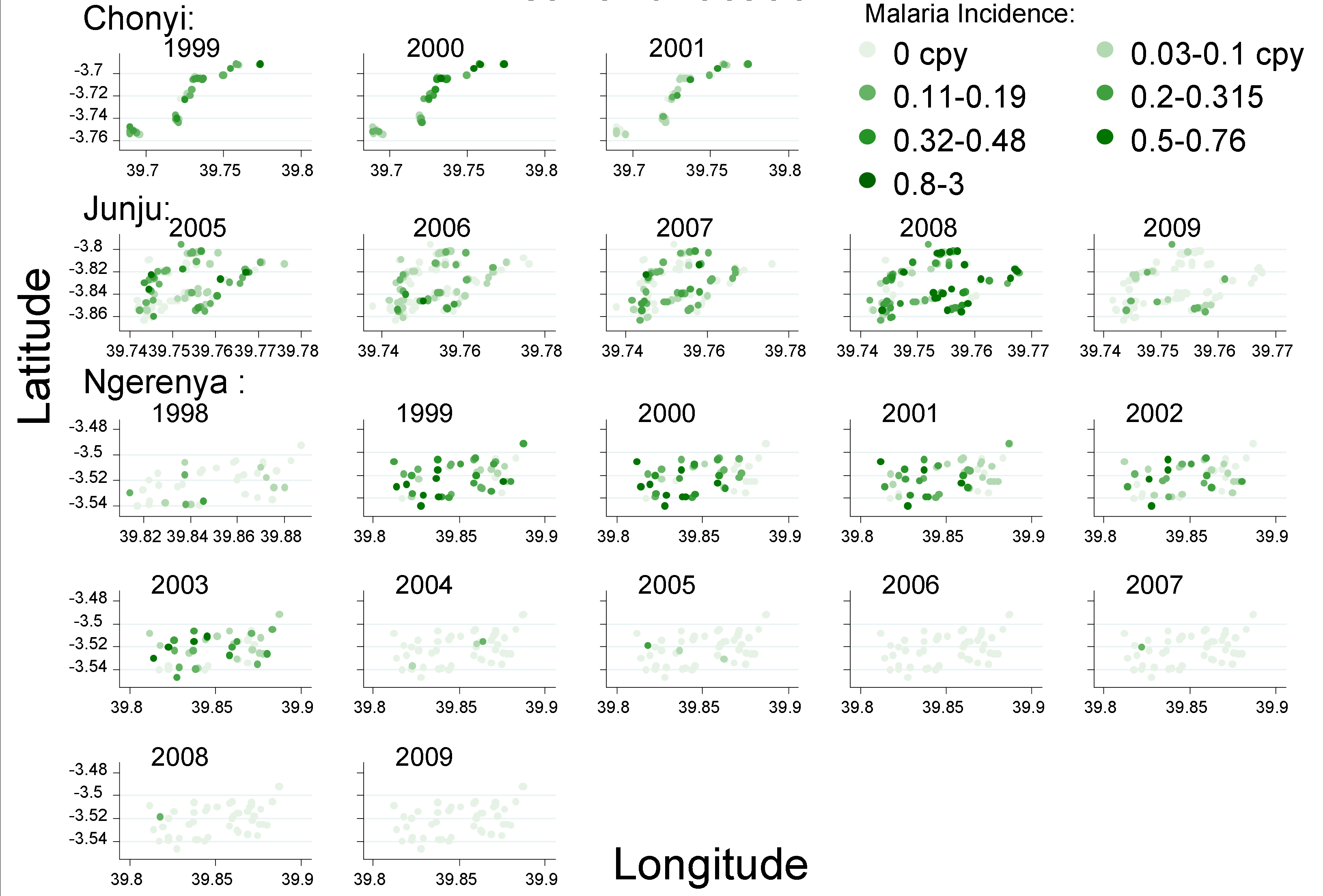

Supplement: Figure S2 — The incidence of febrile malaria episodes cpy is shown for the three cohorts. More intense green colouring indicates higher incidence. (0.48 MB TIF) [file pmed.1000304.s002.tif]

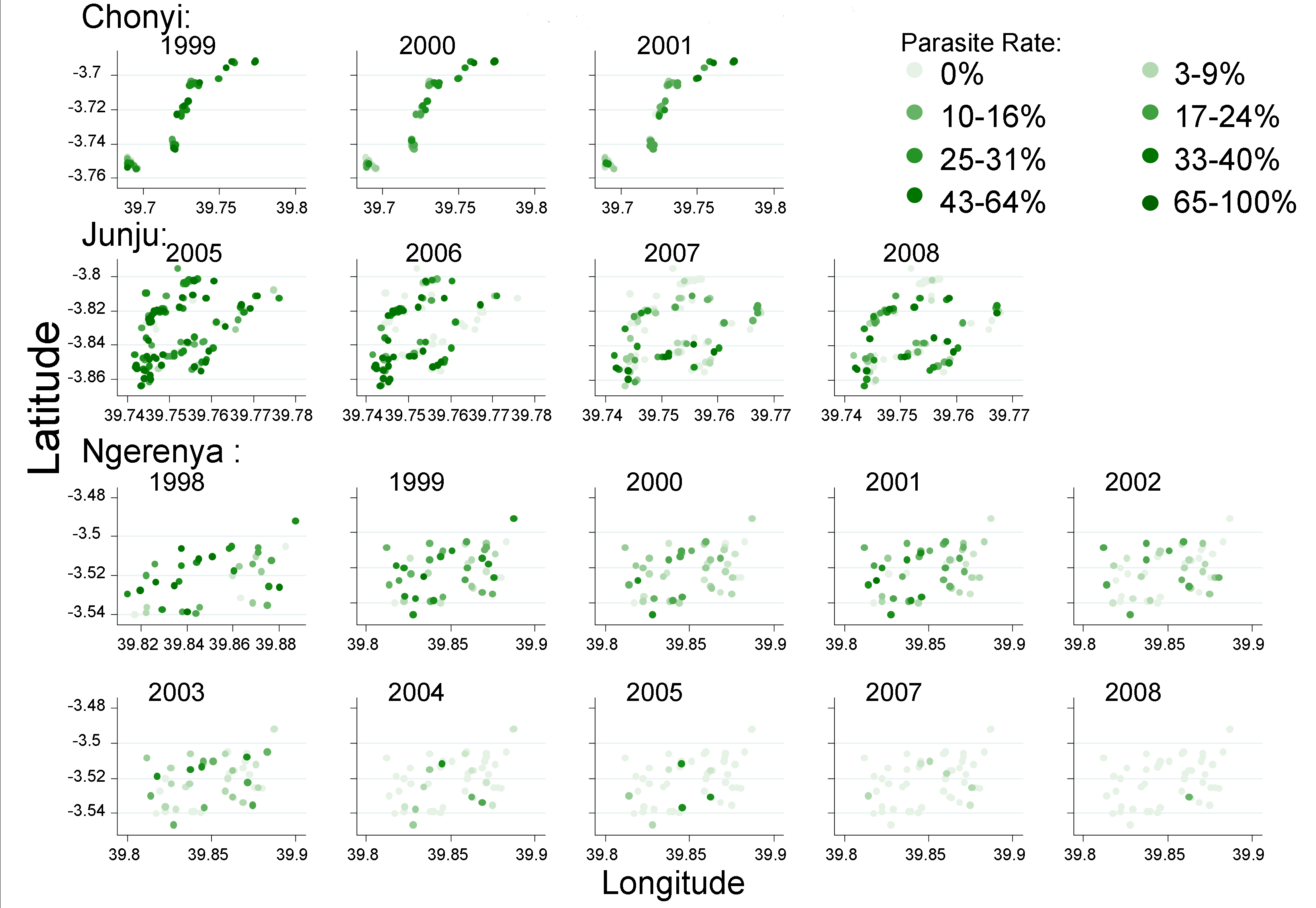

Supplement: Figure S3 — The prevalence of asymptomatic malaria is shown for the three cohorts. More intense green colouring indicates higher incidence. (0.47 MB TIF) [file pmed.1000304.s003.tif]
